# Physicists should perform reference planning for CBCT guided online adaptive radiotherapy

**DOI:** 10.1002/acm2.14163

**Published:** 2023-09-30

**Authors:** Mu‐Han Lin, James A. Kavanaugh, Minsun Kim, Carlos E. Cardenas, Yi Rong

**Affiliations:** ^1^ Radiation Oncology University of Texas Southwestern Medical Center Dallas Texas USA; ^2^ Radiation Oncology Mayo Clinic Rochester Minnesota USA; ^3^ Radiation Oncology University of Washington Seattle Washington USA; ^4^ Radiation Oncology University of Alabama at Birmingham Birmingham Alabama USA; ^5^ Radiation Oncology Mayo Clinic Phoenix Arizona USA

## 1 INTRODUCTION

Radiotherapy (RT) is at crossroads, as the technological improvements are pivotal in improving its treatment efficacy. This presents a paradoxical question: are the people driving the technology, or is it the technology driving the people in this field? In the recent years, one of the advancements that draw the most attention is the online adaptive radiotherapy (oART), which is known for its time consuming and resource intensive nature. As literature continues to pour in regarding knowledge sharing on how to best utilize this technology in terms of workflow, personnel training, and resources allocation, it is evident that a lot of critical steps in this process require expert attention. Even though commercial platforms largely streamline the process and build in automation for efficiency, there are still multiple steps that need to be performed manually or require further exploration. Amongst these steps, the reference planning specifically designed for oART is a critical step to ensure high quality and efficient treatment for each adaptive fraction. A contentious debate arises who should shoulder the responsibility in the reference planning for each oART session? Herein, we have invited a panel of medical physicists from four major institutions who are experts in oART to discuss their experience in building the oART workflow. The proposition for this parallel‐opposed debate is “Physicists should perform reference planning for cone beam CT (CBCT) guided online adaptive radiotherapy.” Arguing in favor of the proposition, we have Dr. Mu‐Han Lin and Dr. James Kavanaugh. On the opposing side, arguing against the proposition, we have Dr. Minsun Kim and Dr. Carlos E. Cardenas.

Dr. Mu‐Han Lin is an associate professor and the Director of Treatment Planning at the Department of Radiation Oncology, University of Texas Southwestern Medical Center. Dr. Lin also serves as the lead physicist of Ethos and Unity adaptive therapy service. Her research focus is translating and implementing artificial intelligence models and automations to improve clinical workflow. She and the team at UTSW have successfully implemented the AI malignancy prediction, AI synthetic CT, AI segmentation, and AI dose prediction modes into routine clinical use. She is also actively involved in the society such as the AAPM working group on treatment planning (WGTP) and the Task Group No. 395.

Dr. James Kavanaugh is an Assistant Professor of Medical Physics at Mayo Clinic in Rochester, Minnesota. His clinical focus is leading the implementation and growth of the online adaptive radiotherapy program utilizing two CBCT‐based Varian Ethos platforms. Research interests focus on the development of novel treatment delivery techniques, including spatial fractionation, knowledge‐based planning, adaptive radiotherapy, and multi‐criteria optimization, and the translation of these techniques to clinical practice. He is a member of the AAPM's Working Group on Treatment Planning and serves on AAPM Task Group No. 308.

Dr. Minsun Kim received her Ph.D. degree in Applied Mathematics from the University of Washington (UW). Currently, she is an Associate Professor at UW and serves as the Clinical Physics Lead at the UW Medical Center. Her research focuses on the spatiotemporally optimal design of radiation treatment planning for better clinical outcomes and her clinical interests include adaptive radiotherapy and automation to improve patient safety and workflow efficiency. She is involved in the professional activities of the American Board of Radiology, American Association of Physicists in Medicine, The Radiosurgery Society, and NRG Oncology. She also serves as Physics Section Editor in Practical Radiation Oncology.

Dr. Carlos E. Cardenas received his PhD degree in Medical Physics from The University of Texas MD Anderson Graduate School of Biomedical Sciences. Currently, he is an Assistant Professor and Director of Automated Treatment Planning in the Department of Radiation Oncology at the University of Alabama at Birmingham (UAB). His research laboratory focuses on leveraging artificial intelligence and automation to improve the quality of radiation oncology care. His clinical interests include the translation and evaluation of novel clinical automated solutions such as those used for adaptive radiotherapy. He provides clinical coverage for UAB's adaptive radiotherapy service, which utilizes the Varian Ethos treatment unit for online adaptive radiotherapy treatment delivery. He is a member of the AAPM's Working Group on Treatment Planning and serves on AAPM Task Groups No. 384 and No. 395 which are tasked with developing recommendations for online adaptive radiotherapy.

## 2 OPENING STATEMENT

### 2.1 For the proposition: Dr. Mu‐Han Lin and Dr. James Kavanaugh

Recent advancements in online imaging, AI‐generated contouring, and auto‐planning in RT have enabled the creation and successful clinical adoption of an efficient CBCT based oART platform.^1^, ^2^, ^3^, ^4^ This oART process requires the creation of a reference plan generated on the traditional simulation CT and baseline anatomical contours. During this reference planning stage, a treatment planner is responsible for setting the formulas for margin expansion, deriving planning structures and optimization parameters. Subsequently, the oART process will use these predefined parameters from the reference plan to generate contours, automatically derive planning structure, and re‐optimize the plan during each oART treatment fraction.

Medical physicists’ responsibilities for a traditional linear static RT workflow include quality control checks (plan check, patient specific QA, weekly chart checks) throughout the planning and treatment process.^5^ Once a plan is approved, the treatment delivery accuracy falls on the attendings and radiation therapists through the on‐board image matching and patient positioning process. This compartmentalized approach, bifurcating the treatment planning and treatment delivery, is not feasible within the CBCT‐guided oART process, as the parameters used to create a reference plan has an intended direct impact on the quality of the available adaptive plans for each individual fraction. Therefore, it is crucial for the oART team to have a comprehensive understanding on both the treatment optimization and the adaptive plan creation when they need to make decisions on selecting and executing high quality treatment plans. Within the oART team, the physicist is the lone member with the technical knowledge, clinical treatment planning skillset, and innovative problem‐solving expertise needed to ensure the quality of the adaptive reference plan. As such, we hold the position that the radiation oncology physicist should be responsible for generating the reference plan for CBCT‐guided oART at the current stage.

Medical physicists have the technical and planning experience required for the creation of quality baseline reference plans within the Ethos treatment planning environment. Initially, the intelligent optimization engine (IOE) in the Ethos TPS has a relatively steep learning curve for planners. Most of the optimization tools available in traditional treatment planning systems are hidden within the IOE and a planner only has indirect control of the process by modifying the ranking of desired dosimetric goals.^6^, ^7^ Good understanding on how the IOE translates ranked goals into a desired treatment plan requires clinical experience and practice using this new technology. Physicists have the best technical knowledge of the IOE and its performance on the resulted oART plans.

Generating an oART reference plan requires additional considerations from planners, medical physicists are well‐equipped with the clinical and physics considerations for online adaptive plan robustness. The reference plan for the CBCT‐based oART should be robust against the potential changes in anatomy to ensure high quality adapted plan for each fraction. Planners should consider the potential uncertainties and take their impact into account when defining beam angles, derived optimization structures, and non‐conflicting optimization objectives. This forward projected planning strategy is then translated into a clear, patient specific directive for the online adaptive planning. With the utilization of auto‐contouring, there is no more “pushing” structures or volumes that planners can add on the fly since they cannot be easily reproduced during oART. Instead, the planning structures need to be pre‐determined and, ideally, derivable from anatomical structures or targets, allowing the structure to be adjusted automatically in response to anatomy or target changes during the course of treatment. For plan generation, the IOE is driving the optimization based on the ranking of planning goals setup by the planners. These goals need to robust against future potential conflicts, taking into account both planning priorities and the dynamic relationships of organs/targets throughout the course of treatment. The knowledge on how anatomy can change and its associated impact on adaptive plan quality is gained through experience covering online adaptive cases.

The online process is intense and time sensitive, requiring quick informed decisions in response to changing anatomy. Minimizing the variation in contouring/planning can help the team member covering oART to stay focused and avoid the stress of comprehending an unfamiliar planning strategy. Without sufficient experience of interacting with the IOE or detailed knowledge on contouring/optimization objectives used to generate the reference plan, it is difficult to make specific suggestions while at the machine that will result in a quality adaptive plan. Having the physicist creating the reference plan will give them the opportunity for hands on experience to identify the key aspects impacting plan quality and collaborate with physicians to develop the contour, planning, online imaging strategies for each individual treatment site. This kinetic can also stimulate more development ideas to improve the process.

Indeed, the continued development of the CBCT‐based adaptive treatment planning process within the clinic is necessary given the nascent state of the technology and its growing utilization. The technology is constantly being refined, with inclusion of more AI‐powered auto‐contouring, modifications to the optimization engines, improvements in dose calculation efficiency, and changes to automated systems/workflows. Additionally, direct feedback from physicians as their understanding of the tools and outcomes evolve will influence the goals and approach to ART planning. Accounting for rapid changes in both the technological tools and clinical objectives will influence how reference plans are constructed for future patients. Having the physicist as the creator of the reference plan will enable efficient changes to the planning process while simultaneously eliminating the possibility of the coverage team being unaware to these modifications, thus minimizing potential errors that could impact the quality of the plan delivered to the patient.

Finally, the involvement of physicists in planning will help the physicists to better develop the checks to catch potential errors prior to impacting patient's treatments while simultaneously improving overall efficiency. This involvement should include the (1) planning phase: to effectively flag the deficiency in the reference plans and get it corrected in time; (2) physics chart check phase: to ensure the reference plan strikes the key aspects to enable a robust adaptive treatment; and (3) online treatment phase: to efficiently check patient setup, imaging, and contouring are all within the specifications to produce a high quality plan. A physicist with detailed involvement in all three phases will allow the development of a comprehensive series of quality controls that minimize the potential propagation of errors that impact the treatment delivered to the patient.

In summary, the involvement of physicists in all aspects of the process, including reference planning, will ensure the creation of high quality plans in a rapidly changing technical and clinical environment. Additionally, physicists will gain experience through reference plan creation, and then provide feedback to further improve existing planning approaches, overcome challenges, and further advance the field by generating consensus and standardization guidelines in the adaptive planning process. The CBCT‐guided oART is still in its infancy. Once the process is streamlined, other team members, such as dosimetrists, could also take on the reference planning seamlessly when the process reaches its maturity. This will speed up the widespread application of the online adaptive therapy to really change the profile of patient care. Similar successful examples, such as the development of SRS and SAbR, had physicists’ involvement in the planning significantly contribute to the standardization and generalization and inspired the developments in planning tools to make these advanced technologies widely implemented in all practices. Having physicist participating in adaptive strategy design is also in line with the future of medical physicist's role—more front facing to investigate and advocate for improvement to patient care.^5^

### 2.2 Against the proposition: Dr. Minsun Kim and Dr. Carlos E. Cardenas

RT relies on the collaborative efforts of a multidisciplinary team, composed of highly skilled professionals trained in various disciplines, who work together to provide optimal patient care. Within this team, medical physicists in Radiation Oncology play a crucial role in ensuring the comprehensive safety of patients undergoing radiation therapy. Their responsibilities encompass diverse aspects, including quality assurance of treatment equipment, ensuring the high quality of patient‐specific treatment plans, and assuring the safe delivery of treatments. As technology advances and becomes more complex, the role of medical physicists is evolving to adapt to these advancements while upholding patient safety.

Medical physicists serve a key role in the clinical implementation of novel technologies, having the overarching goal of incorporating and overseeing new clinical processes introduced with new technologies while ensuring patient safety. Online ART is no exception. While their expertise and knowledge are essential for the successful implementation of oART, it is important to note that their vital role is in training and supporting other professionals to ensure safe and effective utilization of oART rather than taking over their jobs. When it comes to treatment planning, medical physicists are expected to provide comprehensive training and support to dosimetrists. This involves educating them about the principles and operation of the new technology, its potential applications, and the best practices for its use. Training sessions can include hands‐on workshops, lectures, and case studies that enable dosimetrists to gain a thorough understanding of the new technology. These sessions also focus on addressing any potential challenges or pitfalls that may arise during implementation of an oART program. In addition, physicists provide ongoing support and guidance to dosimetrists as they navigate the complexities of the new technology. Physicists serve as a resource for troubleshooting technical issues, providing expert advice, and ensuring that treatment plans are accurately and precisely executed. By equipping dosimetrists with the necessary knowledge and skills, physicists empower them to acquire proficiency in optimizing patient plans with the new technology.

Upon conducting proper initial training and establishing continuous communication with medical physicists, we believe that dosimetrists are able to develop the necessary expertise to create high‐quality adaptive radiotherapy treatment plans. Their unique skill set and dedicated focus on treatment planning make them well‐suited for the task, ensuring optimal patient outcomes and treatment efficacy. Dosimetrists are highly trained professionals with in‐depth knowledge of treatment planning techniques and software. Certified medical dosimetrists are required to complete the necessary didactics and clinical experience before passing the board exam and take 50 continuing education credits for each 5‐year cycle to maintain their knowledge and stay updated with the latest treatment planning techniques. They closely communicate with radiation oncologists to understand the clinical needs of each patient and this work environment allows them to gather insights from different perspectives and integrate them into adaptive treatment plans. Their capacity to communicate and coordinate with the team ensures that treatment adaptations are seamlessly implemented.

While physicists have diverse responsibilities ranging from quality assurance of treatment equipment to safe delivery of patient plans, dosimetrists are dedicated to treatment planning and therefore have time allocated solely to the intricate task of developing optimal treatment plans. This exclusive focus allows them to devote the necessary time and attention to details, ensuring that each treatment plan is meticulously tailored to the individual patient's needs. Their dedicated time allocation enables them to thoroughly analyze a patient's clinical background, perform quality assurance checks, and collaborate with other clinical team members to refine and optimize treatment plans. By having this dedicated time, dosimetrists are also responsible for staying updated on any last‐minute changes in the patient's treatment plan, as failure to incorporate these changes in a timely manner can potentially harm the patient.

We believe that the collaborative relationship between medical physicists and dosimetrists allows them to further enhance their expertise and develop a deeper understanding of the technology's intricacies. By focusing on training dosimetrists instead of taking over their responsibility, medical physicists create a synergistic environment where multiple disciplines work together to leverage the benefits of new technologies. This approach not only ensures the efficient and effective integration of the technology into clinical practice but also promotes professional growth and development among the technical staff. Physicists can empower dosimetrists to take an active role in the treatment planning process and foster collaboration between the two disciplines, especially when it is a challenging process like online adaptive radiotherapy. This collaborative approach enhances the overall quality of patient care and ensures that the treatment plans are optimized for each individual patient.

We believe that the most effective approach to ensuring patient safety and achieving high quality treatment plans lies in the synergistic effort among medical physicists, dosimetrists, and physicians. By leveraging the expertise and contributions of each team member, we can optimize patient care. This collaborative effort, where each professional performs at their best, leads to enhanced patient safety and improved clinical outcomes.

## 3 REBUTTAL

### 3.1 For the proposition: Dr. Mu‐Han Lin and Dr. James Kavanaugh

First, we would like to start with the points from our opponents that we agree with. Medical physicists play a crucial role in the clinical implementation of novel procedures. Lectures, hands‐on workshops, and case studies on CBCT‐based oART are good educational opportunities for dosimetrists and other personnel involved. Additionally, we are in an agreement that a close partnership between physics and dosimetry during the planning process will leverage the expertise of both parties in developing customized patient specific treatment plans. However, we argue for the unique responsibilities and vital roles of medical physicists throughout the planning and delivery of oART. Specifically, the critical knowledge and practical understanding on creating adaptive plans from reference plans in daily adaptive session requires medical physicists to develop and maintain hands‐on experience with the auto‐planning features in the CBCT based oART system. Unlike conventional planning that is well studied with a wealth of knowledge in literature, the CBCT‐based oART planning, as an emerging technology, still has many clinical aspects being investigated and may present significant challenges to staff members who are not as experienced as well‐trained medical physicists. Even for medical physicists with extensive experience in the conventional planning, the auto‐planning features in the CBCT‐based oART TPS are unique and require specialized training. Hence, the hands‐on planning experience with this system is necessary for a medical physicist to better oversee the on‐treatment procedure and navigate the auto‐planning, for which the planner cannot see the hyper‐parameters for optimization.

The goal of medical physicist performing reference planning at the current phase is NOT to take over dosimetrists’ job. Instead, the goal is to develop a stronger collaboration between medical physicists and dosimetrists during the ongoing implementation and advancement CBCT‐based oART. As our opponents mentioned, dosimetrists are highly trained professionals and with proper guidance, they will be able to perform the reference planning very well. However, CBCT‐based oART introduces brand new auto‐planning concepts and tools that do not allow an easy adoption even with the existing skillsets and expertise in dosimetry. In‐depth understanding of the reference plan and adaptive optimizer when applying to anatomies visualized on a simulation CT and potential changes present on daily CBCT is critical for the physicist covering the adaptive fractions, especially when plan modification is deemed necessary due to anatomic changes. Traditional approach involves extensive commissioning, planning studies, and protocol development before implementation of technology. Extensive resources would be required for oART protocol development following this retrospective development approach, since one would need to emulate the oART contour‐to‐plan generation process to evaluate the effectiveness of an approach. Physicists’ direct prospective involvement in the reference planning is a more efficient and effective to build robust adaptive protocols. Training dosimetrists on standardized protocols would be the next step.

Our opponents have argued that one important role of the dosimetrist is to closely work with the radiation oncologist to translate their stated clinical goals into a treatment plan. They can receive direct feedback from the physician and make last minute changes, allowing for a tailored plan that meets each patient's needs. Within the traditional non‐ART workflow, the dosimetrist's role is ideally positioned to fulfill these stated goals. However, within the oART workflow the close collaboration between physician and planner occurs while treating the adaptive fractions. Feedback from the physician on how the observed changing anatomy and impact on the achieved adaptive plan inform a continual improvement for future planning. In most institutions, it is the physicist, not dosimetrist, who is physically present at each adaptive fraction and can translate this feedback from the physician into changes in the planning process.

Both sides agree that physicist's involvement in adaptive therapy planning is crucial. Our opponents mentioned that physicists can get involved early in checking the adaptive intent, contouring and deriving structure setup before planning and review planning strategies before the physician's plan review. These are certainly helpful. However, this will add to the turnaround time from simulation to treatment start. With their proposed process, how many institutions can afford to add these extra hands‐off steps during planning prior to physician review? And again, without practical knowledge about the TPS, how effective can a physicist be to identify the pitfalls of dosimetrist’ planning strategy? Would ineffective guidance and multiple iterations of planning hurt the trust between dosimetrists and physicists? If a poorly constructed reference plan made it to the on‐treatment adaptive fractions, it will pose concern of a lower quality plan being generated and the covering team would either need to abort the treatment, accept the lower quality plan, or implement some change at the treatment machine to hopefully improve the plan. If the physicists are not familiar with the optimizer of the system, how can physicists be able to oversee or direct the oART process and troubleshoot in real time? For example, in the scenario in which the first attempt of oART did not generate an acceptable plan, should a second attempt be pursued? And what changes can be applied to make the second attempt successful?

In conclusion, we stand firmly on our proposition that physicists should perform reference planning for CBCT guided oART at the current phase. A partnership with dosimetry while generating reference plans will allow for the physicist to maintain their knowledge and skills associated with the plan creation process, incorporate dosimetry training, and insights into the development of oART treatment protocols. As the oART technology and workflow mature, a transition of planning from physics to dosimetry can occur for sites and protocols that have been well established. Additionally, medical physicists can use the in‐depth knowledge and practical experience gained from simulation to treatment of oART to assist clinical operation in their institution and further pass on to other institutions who are adopting oART.

### 3.2 Rebuttal against the proposition: Dr. Minsun Kim and Dr. Carlos E. Cardenas

We agree that it is crucial for the oART team to have a comprehensive understanding of the treatment planning and adaptive plan creation processes. We also agree that the physicist, being a member with technical knowledge and expertise, plays a pivotal role in ensuring the quality of the reference plan. However, this does not necessitate the physicist taking over the role of creating the reference plan, nor does it imply that the physicist's plan results in superior quality.

Unlike adaptive plans for treatment sessions with time constraints, the reference plan is crafted with ample preparation time and sufficient resources available to the planner. In such an environment, integrating diverse perspectives and viewpoints from both disciplines, dosimetrists and physicists, can improve the quality of the plan and patient safety. This approach stands in contrast to bypassing one discipline (dosimetrists) recognizing that both physicists and dosimetrists contribute distinct perspectives and strengths to patient care. The proposition argues that physics oversight of planning ensures proper definition of contours, derivations, and planning goals. Here, we counterpoint that physics’ review of predefined patient‐specific planning parameters (contours, derivations, goals, etc.) could be performed prior to planning commencement to prevent any delays. Intent reviews could be implemented in the planning workflow to ensure adequate preparation prior to plan optimization within the IOE.^8^

We agree that the planning techniques and optimization tools utilized within the IOE differ significantly from those in traditional TPS. However, our perspective maintains that the duty to impart knowledge and expertise to dosimetrists lies with the medical physicist, rather than assuming the role of primary planners. While physicists may possess a deeper comprehension of the underlying optimization algorithms and hidden logics within the Ethos TPS, it is essential to acknowledge that dosimetrists possess a broader range of day‐to‐day planning experiences, encompassing diverse patient anatomies and clinical workflows. Furthermore, medical physicists can contribute by developing well‐curated planning templates that dosimetry can use as starting points in the planning process. Several groups have published and shared their experience developing Ethos TPS planning templates,^1^, ^7^, ^9^, ^10^, ^11^, ^12^, ^13^, ^14^ which promise to further optimize the planning process and improve plan quality. Making these planning templates opened‐access and sharable for multiinstitutional evaluation and validation could further enhance medical physicists’ role in adaptive treatment planning,^15^ leading to a streamlined implementation of planning templates by dosimetry.

Patient safety is also reinforced to a certain extent through repetitive experiences, rather than solely relying on the depth of theoretical knowledge. If an institution hires medical physicists whose primary responsibility is treatment planning, this could be a viable option. However, in the majority of institutions, medical physicists have diverse responsibilities encompassing various aspects of patient care. They may not be as adept as dosimetrists in the day‐to‐day manual tasks, which can potentially compromise patient safety if not executed accurately. We are of the opinion that there are limitations to physicists assuming treatment planning responsibilities, particularly in terms of resource allocation and patient safety, if the physicist is not fully dedicated to the planning process.

We fully acknowledge that the forward‐projected planning strategy required for a reference plan to remain robust against future treatment adaptations is important in crafting a reference plan. The aspect of an online adaptive planning platform with its restriction demands meticulous consideration during the reference plan's inception. Given that this concept is novel to both physicists and dosimetrists, it accentuates the necessity for collaborative efforts between these two disciplines, each contributing their unique strengths. This reiterates the importance of prior training before implementing new technology in clinical settings. Collaborating with institutions that are early adopters of novel technologies and transferring that knowledge to one's own institution and staff forms a crucial component of physics commissioning.

We align with the viewpoint of Drs. Lin and Kavanaugh regarding the significance of incorporating knowledge from the reference plan when devising adaptive plans under time pressure. Equally valuable is the knowledge that physicists gain during the adaptive planning process at the machine, which contributes to reference plan design. This underscores the utmost importance of collaborative teamwork for the success of the online adaptive radiotherapy program. The establishment of a robust feedback loop between physicists and dosimetrists stands as the cornerstone in the implementation of oART for enhanced patient care. This feedback mechanism underscores the strong working rapport among the crucial team members. When established effectively, it fosters a more robust environment for advancing the field and, ultimately, improving patient care.

## CONFLICT OF INTEREST

The authors declare no conflicts of interest.
